# Hypoxia and Human Genome Stability: Downregulation of BRCA2 Expression in Breast Cancer Cell Lines

**DOI:** 10.1155/2013/746858

**Published:** 2013-09-22

**Authors:** Daniele Fanale, Viviana Bazan, Stefano Caruso, Marta Castiglia, Giuseppe Bronte, Christian Rolfo, Giuseppe Cicero, Antonio Russo

**Affiliations:** ^1^Section of Medical Oncology, Department of Surgical, Oncological and Stomatological Sciences, University of Palermo, 90127 Palermo, Italy; ^2^Phase I-Early Clinical Trials Unit, Oncology Department and Multidisciplinary Oncology Center Antwerp (MOCA), Antwerp University Hospital, 2650 Edegem, Belgium

## Abstract

Previously, it has been reported that hypoxia causes increased mutagenesis and alteration in DNA repair mechanisms. In 2005, an interesting study showed that hypoxia-induced decreases in BRCA1 expression and the consequent suppression of homologous recombination may lead to genetic instability. However, nothing is yet known about the involvement of BRCA2 in hypoxic conditions in breast cancer. Initially, a cell proliferation assay allowed us to hypothesize that hypoxia could negatively regulate the breast cancer cell growth in short term in vitro studies. Subsequently, we analyzed gene expression in breast cancer cell lines exposed to hypoxic condition by microarray analysis. Interestingly, genes involved in DNA damage repair pathways such as mismatch repair, nucleotide excision repair, nonhomologous end-joining and homologous recombination repair were downregulated. In particular, we focused on the BRCA2 downregulation which was confirmed at mRNA and protein level. In addition, breast cancer cells were treated with dimethyloxalylglycine (DMOG), a cell-permeable inhibitor of both proline and asparaginyl hydroxylases able to induce HIF-1**α** stabilization in normoxia, providing results comparable to those previously described. These findings may provide new insights into the mechanisms underlying genetic instability mediated by hypoxia and BRCA involvement in sporadic breast cancers.

## 1. Introduction

Breast cancer (BC) is one of the leading causes of cancer-related death among women worldwide [1]. About 5–10% of familial breast cancers can be attributed to two autosomal dominant genes with high penetrance: *BRCA1* and *BRCA2* [2]. Carriers of germline mutations in *BRCA1* and *BRCA2* have a predisposition for developing breast and/or ovarian cancer [3]. In addition, it has been reported that *BRCA1* expression was reduced or undetectable in the majority of high-grade, ductal carcinomas, suggesting that absence of *BRCA1* may contribute to the pathogenesis of a significant percentage of sporadic breast cancers [4, 5]. BRCA plays an important role in DNA repair, activation of cell-cycle checkpoints, and maintenance of chromosome stability [6, 7]. In the last years, several authors reported that the tumor microenvironment can contribute to genetic instability and alter the overall DNA repair [8–14]. 

Mammalian cells are extremely intolerant to prolonged exposure to hypoxia; contrariwise tumor cells are tolerant to anoxia and many tumors contain hypoxic regions [15, 16]. Intratumoral hypoxia is an adverse clinical prognostic factor associated with decreased disease-free survival for many cancers such as the prostate, cervix, breast, and head and neck [17–19]. Hypoxic tumor cells can be locally and systematically aggressive with a decreased sensitivity to apoptotic and other cell death signals, increased angiogenesis, increased proliferation, and increased capacity for systemic metastasis [20, 21]. It is now well known that hypoxia causes the stabilization of HIF-1*α* monomer that translocates to the nucleus where it heterodimerizes with HIF-1*β* and HIF-1 complex binds to the hypoxia responsive element (HRE) on the promoter regions of target genes in order to promote tumor survival, invasion, and metastasis [22–25]. Recently, it has been shown that under severe hypoxia conditions the mismatch repair (MMR) genes, *MLH1* and *MSH2*, are downregulated in p53- and HIF-1a-dependent way in many tumor cell lines, thus inducing genetic instability [10, 26]. Furthermore, Mihaylova et al. reported that cells exposed to hypoxia- and low pH were found to have a diminished capacity for DNA repair compared with controls [26]. In 2005, an important study showed that hypoxia-induced decreases in *BRCA1* expression and the consequent suppression of homologous recombination may lead to genetic instability [27]. However, nothing is yet known about the involvement of *BRCA2* in hypoxic conditions in breast cancer.

Here, we aim to analyze gene expression in breast cancer cell lines exposed to hypoxic condition with a focus on genes involved in *DNA damage repair* (DDR), especially *BRCA2*. 

## 2. Materials and Methods

### 2.1. Cell Cultures

Human BC cell lines, MCF-7, MDA-MB-231, and SKBr3, purchased from the American Type Culture Collection (Rockville, MD, USA), were cultured in Dulbecco's Modified Eagle Medium (DMEM : F12) supplemented with 10% fetal bovine serum (FBS) and antibiotics (100 U/mL penicillin and 50 mg/mL streptomycin) (Invitrogen, Carlsbad, CA, USA). Eighty percent of confluent cell lines were cultured in normoxic atmosphere of 16% O_2_, 79% N_2_, and 5% CO_2_ (by volume) for 24 h. Then medium was changed and cells were further cultured under normoxia or hypoxia (3% O_2_, 87% N_2_, 5% CO_2_, by volume) at two different time-points, 24 h and 48 h. Furthermore, cells were incubated in the absence (normoxia) or presence (hypoxia) of HIF hydroxylase inhibitor, dimethyloxalylglycine (DMOG) (Sigma-Aldrich, St. Louis, MO, USA), for 24 h and 48 h, at final concentration of 1 mM.

### 2.2. Cell Proliferation Assay

To analyze cell proliferative activity, a 5-bromo-2′-deoxyuridine (BrdU) assay was performed using BrdU Cell Proliferation Assay Kit according to the manufacturer's instructions (Cell Signaling Technology, Euroclone, Milano, Italy). Cells were seeded at 1 × 10^4^ cells/well in a 96-well plate and incubated overnight. To assess proliferative activity under hypoxia, cells were directly incubated under normoxia and hypoxia (for 24, 48, 72, and 96 hours). Ten *μ*M BrdU was added to the plate, and the cells were incubated for 4 hours. The absorbance at a wavelength of 450 nm was measured using an ELISA microplate reader. All experiments were performed in triplicate.

### 2.3. Microarray Analysis

Microarray analysis was performed as previously described [28]. Total RNA was extracted according to the manufacturer's protocol (Affymetrix, Santa Clara, CA, USA). Fragmented cRNA was hybridized using a human oligonucleotide array U133 Plus 2.0 (Genechip Affymetrix, Santa Clara, CA, USA). Washing and staining were performed through Affymetrix GeneChip Fluidic Station 450. Probe arrays were scanned using Affymetrix GeneChipScanner3000 G7enabled for high-resolution scanning. Two biological replicates were performed for each experimental condition. Images were extracted with the GeneChip Operating Software (Affymetrix GCOS v1.4). Quality control of the arrays was performed using the AffyQCReport software [29]. 

### 2.4. Statistical Analysis

For statistical analysis, the background subtraction and normalization of probe set intensities were performed using the method of robust multiarray analysis (RMA) described by Irizarry et al. [30]. To identify DEGs, gene expression intensity was compared using a moderated *t*-test and a Bayes smoothing approach developed for a low number of replicates [31]. To correct for the effect of multiple testing, the false discovery rate was estimated from *P* values derived from the moderated *t*-test statistics [32]. The analysis was performed using the affylmGUI Graphical User Interface for the limma microarray package (Bioconductor Software) [33]. Significant differences were determined by Student's *t*-test. *P* value <0.05 was considered to be statistically significant. 

### 2.5. Microarray Data Analysis

Hierarchical cluster and heat map analyses (HCA) were performed using the MultiExperiment Viewer (MeV v4.8) program of TM4 Microarray Software Suite. Gene Set Analysis Toolkit was used to investigate the biological significance of a set of genes represented by the specific expression pattern in DNA repair mechanisms. DEGs were analyzed according to predefined pathways annotated by KEGG [34] and Biocarta bioinformatic resources. For an overrepresented KEGG or Biocarta pathway, a cut-off *P* value of 0.01 was selected. All showed values are in logarithm scale.

### 2.6. Quantitative Real-Time PCR (qRT-PCR) and RT-PCR

Total cellular RNA was extracted using RNeasy Mini Kit (Qiagen Inc., Valencia, CA, USA). Then, RNA was controlled through 2100 Bioanalyzer (Agilent Technologies, Santa Clara, CA, USA) and quantified through the spectrophotometer NanoDrop ND-1000 (CELBIO). For *BRCA1*, *BRCA2,* and *MLH1* mRNAs detection, 2 ng of total RNA was reverse transcribed into single-stranded cDNA using High Capacity cDNA Reverse Transcription Kit (Applied Biosystems, Foster City, CA, USA) according to vendor's instructions. Gene-primers for *BRCA1*, *BRCA2,* and *MLH1* were purchased from Applied Biosystems (TaqMan gene expression assay). Quantitative real-time PCR (qRT-PCR) was performed with the ABI PRISM 7900 sequence detection system (Applied Biosystems, Foster City, CA, USA) using SDS software version 2.1. The reactions were performed in triplicate and the results were normalized using Human *β*-actin Predeveloped TaqMan assay reagents (Applied Biosystems). Changes in the target mRNA content were determined using a comparative CT method (ABI User Bulletin number 2). An average CT value for each RNA was obtained for triplicate reactions. 

### 2.7. Western Blotting (WB)

Cells were lysed using complete Lysis-M reagent set (Roche, Mannheim, Germany). Protein concentration was measured using Quick Start Bradford (Bio-Rad Laboratories, Hercules, CA, USA). 80–100 mg of total protein lysate was separated on 10% polyacrylamide gel under denaturing conditions and immunoblotted into nitrocellulose membrane. The following antibodies (Abs) were used: anti-BCRA2 goat (Santa Cruz Biotechnology, Santa Cruz, CA, USA); anti-GAPDH(6C5) (Santa Cruz Biotechnology, Santa Cruz, CA, USA); anti-HIF-1a rabbit (Bethyl Laboratories, Montgomery, USA).

## 3. Results

### 3.1. Effects of Hypoxia on Cell Proliferation

**Figure 1:**
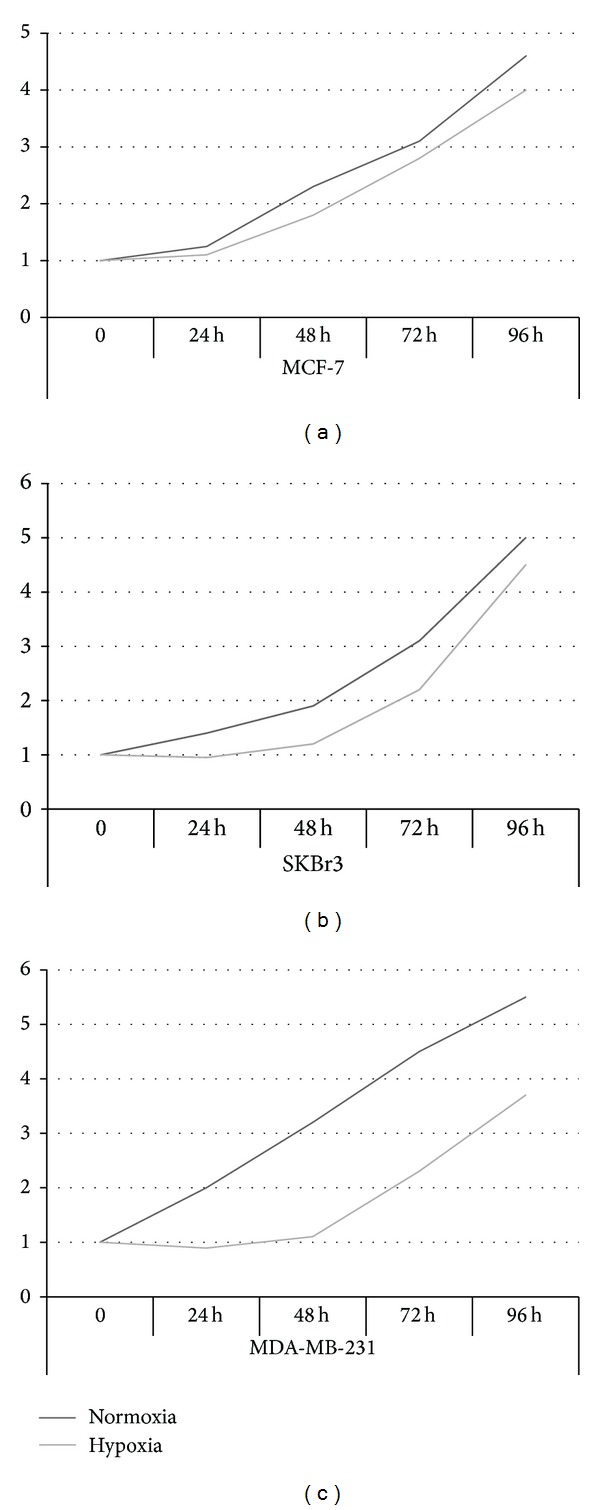
Role of hypoxia in cell proliferation. Cell proliferative activity was evaluated by BrdU incorporation assay. Data represent means of different culture experiments, each performed in triplicate and are presented as fold changes ± SD. Cell proliferative activity at time 0 was set to 1. MCF-7 (a), SKBr3 (b), and MDA-MB-231 (c) cell lines were cultured under normoxia or hypoxia for 24, 48, 72, and 96 hours.

To analyze the effects of hypoxia on cell proliferation, a BrdU incorporation assay was performed and proliferative activity was evaluated in BC cells incubated under normoxia and hypoxia at different times. Evaluation of BrdU incorporation percentage showed that hypoxia reduces cell proliferation in MCF-7, SKBr3, and MDA-MB-231 cells (Figure 1). It is interesting to note that hypoxia appears to have a greater effect on proliferation in the first 48 hours. The percentage increase, in fact, after 48 hours appears to be comparable in both conditions (normoxia and hypoxia) in all BC cell lines.

### 3.2. Gene Expression Profiling in Breast Cancer Cells under Hypoxia

Since hypoxia is a condition which during tumor growth influences the expression of several genes involved in angiogenesis, proliferation, cell cycle control, and DNA damage repair (DDR) mechanisms, we first performed a microarray analysis, using Affymetrix platform, in order to compare differential gene expression profiles in MCF-7, MDA-MB-231, and SKBr3 human breast cancer cell lines in response to hypoxic exposure for 24 and 48 hours, respectively. This work was carried out in order to evaluate the involvement of some genes in molecular pathways related to tumor pathogenesis. This analysis has allowed to obtain, for each examined BC cell line, two lists of differentially expressed genes (DEGs) in hypoxia for 24 and 48 hours compared to normoxia (control). In particular, for this study, the lists were screened considering as significant only the genes with fold change (*M*)>|0.5| (logarithm scale) and *P* < 0.05. Thus, we obtained six lists of DEGs with respect to normoxia, including, after 24 h of hypoxia, 10,437 genes (5,958 downregulated and 4,479 upregulated) for MCF-7; 11,267 genes (6,801 downregulated and 4,466 upregulated) for MDA-MB-231; and 14,280 genes (8,450 downregulated and 5,830 upregulated) for SKBr3; and, after 48 h of hypoxia, 11,205 genes (5,955 downregulated and 5,250 upregulated) for MCF-7; 12,756 genes (5,656 downregulated and 7,100 upregulated) for MDA-MB-231; and 17.098 genes (9,180 downregulated and 7,918 upregulated) for SKBr3.

Then, six lists of genes were compared with each other and analyzed in order to identify a set of DEGs common to three BC cell lines. Using Venn diagrams, we found 270 shared genes (81 downregulated and 189 upregulated) that showed a gene expression variation in hypoxia (24 h and 48 h) with respect to normoxia. 

### 3.3. Molecular Pathways Deregulated in Hypoxia

A further series of studies was carried out on the microarray analysis data in order to identify any genes of particular interest. Significant gene expression variations were detected in genes involved in angiogenesis, proliferation, cell cycle progression, mitosis, genomic stability, and response to hypoxia. The integrated analysis resulting from KEGG and Biocarta databases allowed us to identify the main molecular pathways altered in BC cell lines after hypoxic exposure for 24 h and 48 h. This analysis indicated that the genes showing a significant variation in expression levels are included in the following pathways: DNA damage repair (37 genes), cell cycle regulation (35 genes), HIF-1*α* network (15 genes), and mitosis regulation (12 genes). A considerable number of genes involved in proliferation and cell cycle control, through the G1/S and G2/M transitions, showed a significantly deregulated expression in all three BC cell lines. Among the DEGs in hypoxia, required for G1/S transition, *CDC6*, *CCND1*, *CCNE2*, and *CDK2 * were downregulated, whereas *CDKN1A* (p21^Cip1^) was upregulated. Instead, *CDC25A *was downregulated in MCF-7 and SKBr3 cells and upregulated in MDA-MB-231 cells. Moreover, we found that in MCF-7 and SKBr3 cells hypoxia induces downregulation of several genes required for G2/M transition, such as *CCNA2*, *CCNB1*, *CCNB2*, *CDC2 *(*CDK1*), and *CDC25C*. The same results were shown by MDA-MB-231 cells under hypoxic conditions for 24 h.

**Figure 2:**
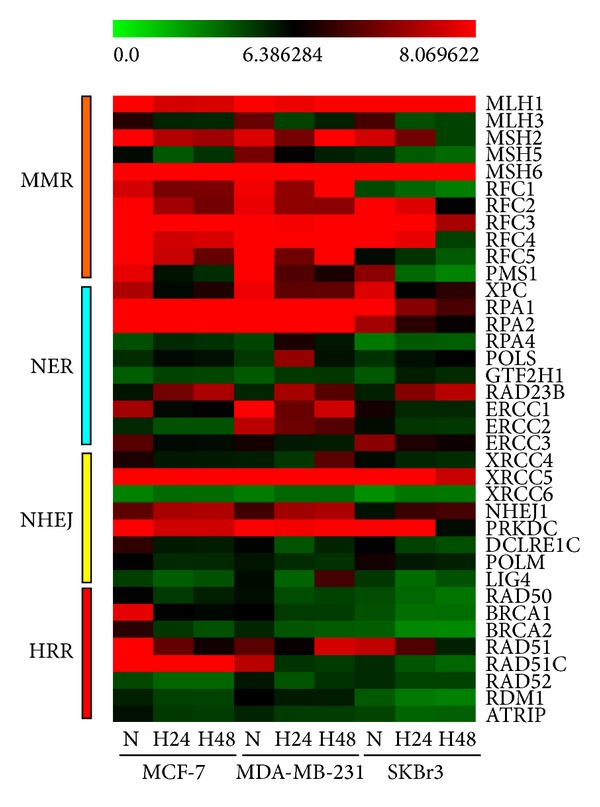
Heat maps of DEGs involved in DNA damage repair (DDR) pathways deregulated in hypoxia. Clustering of DEGs involved in DNA damage repair (DDR) pathways: mismatch repair (MMR), nucleotide excision repair (NER), nonhomologous end-joining (NHEJ), and homologous recombination repair (HRR). The heat maps were generated from microarray data reflecting gene expression values in MCF-7, MDA-MB-231, and SKBr3 cells exposed to hypoxia (3% O_2_) for 24 h and 48 h in comparison to control cells cultured under normoxic conditions (16% O_2_, *M* > |1| and *P* < 0.05). Each row represents the expression levels for a single gene tested for different experimental conditions. Each column shows the expression levels for the genes tested for a single experimental condition. The absolute expression value (log scale) of each gene is derived from the mean of two biological replicates. The color scale bar on the top represents signal intensity variations ranging from green (poorly expressed genes) to red (highly expressed genes). Black boxes indicate intermediate expression values. N = normoxia; H24 = hypoxia for 24 h; H48 = hypoxia for 48 h.

In addition, significant increases in expression levels were also observed in key genes involved in HIF-1*α* network (Figure 2(b)), such as *VEGF-A*, *SLC2A1*, *JUN*, *FOS,* and other genes encoding for glycolytic enzymes (*PGK1*, *PFKFB3*, *HK2*, and *ALDOA*). Finally, microarray analysis showed a significant variation in expression levels of some genes that regulate the mitosis included in AURKA signaling pathway. Other genes involved in this pathway, such as *AURKB*, *JUB* (ajuba), and *TPX2*, are downregulated in all three BC cell lines under hypoxic conditions.

However, since several studies reported that the hypoxia of tumor microenvironment can contribute to genetic instability [35], our attention was focused mainly on the DEGs involved in DNA damage repair (DDR) pathways: mismatch repair (MMR), nucleotide excision repair (NER), nonhomologous end-joining (NHEJ), and homologous recombination repair (HRR) (Figure 2). In general, the expression of genes involved in these pathways was found downregulated (see Table S1 in Supplementary Material available online at http://dx.doi.org/10.1155/2013/746858). The most significant changes were observed in the HRR pathway. In particular, in HRR we observed a significant decrease in expression levels of *BRCA1*, *BRCA2*, *RAD51,* and *RAD50*. Furthermore, there were significant variations in key genes involved in MMR. In particular, we found a significant decrease in the expression levels of *MLH1*, *MSH2,* and *MSH6*. Moreover, we demonstrated a more global effect on DNA damage repair pathways as a result of the hypoxic exposure.

### 3.4. Downregulation of *BRCA2* Expression under Hypoxia

**Figure 3:**
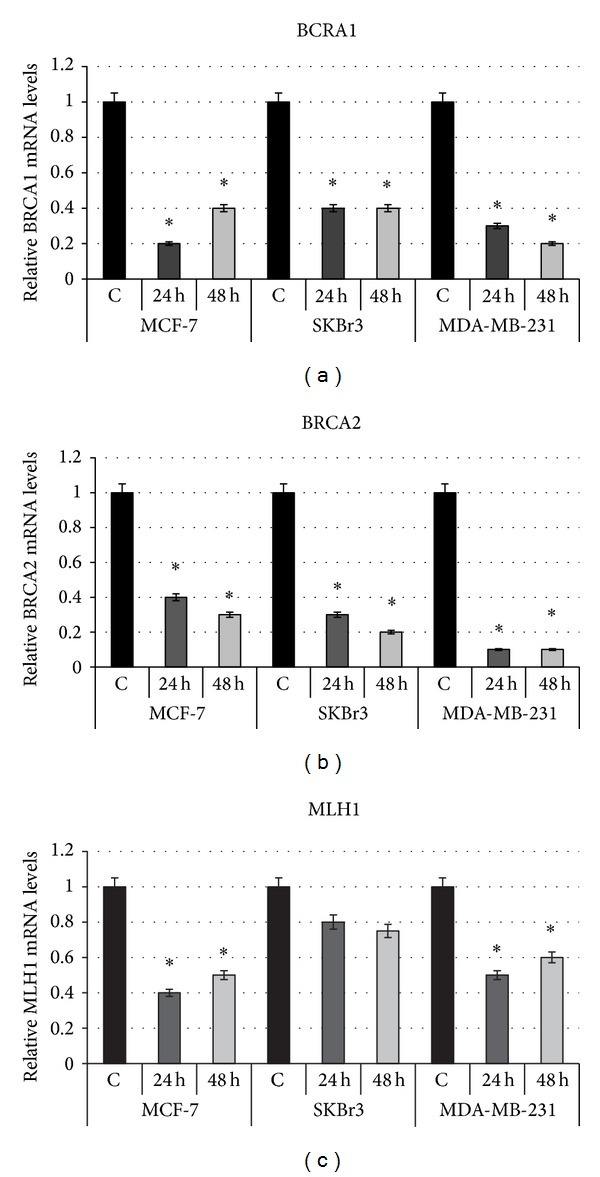
Effects of hypoxia on *BRCA2* expression in breast cancer cell lines. Validation of microarray data by quantitative real-time PCR analysis. Changes in *BRCA1* (a), *BRCA2* (b), and *MLH1* (c) mRNA expression levels were determined in MCF-7, SKBr3, and MDA-MB-231 cells exposed to hypoxia (3% O_2_) for 24 and 48 hours with respect to control condition (normoxia, 16% O_2_). Relative transcript levels were determined using the 2^−ΔΔCt^ method and normalized to *β*-actin mRNA (endogenous control). Normoxic condition values are taken as 1 and hypoxic condition values represent fold decrease relative to control condition. Data are presented as fold changes ± SDs. Significant difference, hypoxia for 24 h (H24) or for 48 h (H48) versus normoxia (N), *P < 0.05.

In order to validate microarray analysis data, we have evaluated the *BRCA2* mRNA expression levels in the same BC cell lines under hypoxic conditions, using qRT-PCR. Moreover, we assessed *BRCA1* and *MLH1* mRNA expression levels, also involved in DNA repair and for which a downregulation in BC cell lines under hypoxic conditions has been already observed. The qRT-PCR analysis confirmed that hypoxia induces *BRCA2 *downregulation in all three BC cell lines used. In particular, *BRCA2 *downregulation was more pronounced in MDA-MB-231 cells (0.1-fold in both conditions); in MCF-7 cells the reduction was 0.4-fold after 24 h of hypoxia and 0.3-fold after 48 h of hypoxia exposure. These results are comparable to those obtained in SKBr3 cells (0.3-fold at 24 h, 0.2-fold at 48 h) (Figure 3(b)). As previously reported, we also observed a downregulation of *BRCA1 *and *MLH1 *mRNA levels under hypoxic conditions (Figures 3(a) and 3(c)). In particular, *BRCA1* was 0.2-fold, 0.4-fold, and 0.3-fold after 24 hours of hypoxia in MCF-7, SKBr3, and MDA-MB-231, respectively, and 0.4-fold, 0.4-fold, and 0.2-fold after 48 hours of hypoxia in MCF-7, SKBr3, and MDA-MB-231, respectively (Figure 3(a)). *MLH1* was 0.4-fold, 0.8-fold, and 0.5-fold after 24 hours of hypoxia in MCF-7, SKBr3, and MDA-MB-231, respectively, and 0.5-fold, 0.75-fold, and 0.6-fold after 48 hours of hypoxia in MCF-7, SKBr3, and MDA-MB-231, respectively (Figure 3(c)).

**Figure 4:**
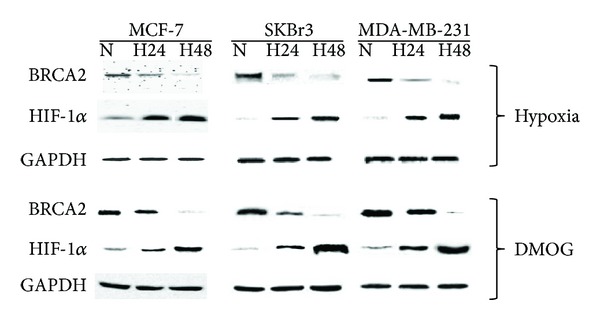
Effects of hypoxia on *BRCA2* and HIF-1*α* protein expression levels in MCF-7, SKBr3, and MDA-MB-231 cells. Protein expression was examined by western blot analysis with the indicated antibodies, using the enhanced chemiluminescence reagent (ECL). The GAPDH house-keeping protein was used as loading control (N = normoxia; H24 = hypoxia for 24 h; H48 = hypoxia for 48 h). The experiments were performed at least three different times. MCF-7, SKBr3 and MDA-MB-231 cells were also incubated with 1 mM DMOG for 24 h (H24) and 48 h (H48).

Interestingly, in parallel to *BRCA2 *mRNA downregulation, we observed, through western blot (WB) analysis, a reduction of *BRCA2* protein levels in MCF-7, SKBr3, and MDA-MB-231 cells after 24 h and 48 h of hypoxia, compared to control condition (normoxia) (Figure 4). HIF-1*α* protein levels were also evaluated. At the same time, BC cells were treated with dimethyloxalylglycine (DMOG), a cell-permeable inhibitor of both proline and asparaginyl hydroxylases able to induce HIF-1a stabilization in normoxia. Thus, treatment of MCF-7, MDA-MB-231, and SKBr3 cells with a PHD inhibitor (for 24 h and 48 h) caused the activation of the HIF-1 pathway in normoxia. In fact, WB analysis provided results comparable to those previously described, leading to an increase in HIF-1*α* expression levels and concomitant reduction of *BRCA2* expression in presence of DMOG (Figure 4). Taken together these data suggest that hypoxia could be the main reason for *BRCA2* downregulation. 

## 4. Discussion

Breast cancer is the most common malignancy of the mammary gland and its incidence increases with age. Nowadays, although oncologists have several available options (chemotherapy, hormone therapy, and biologic agents such as antiangiogenic and anti-HER2 drugs), BC is still responsible for a significant percentage of cancer deaths in women [1, 36]. 80–85% of breast cancer is sporadic, while 15–20% shows a familial history. About 5–10% of cancers can be attributed to two autosomal dominant genes with high penetrance: *BRCA1* and *BRCA2*. *BRCA2* is a protein of about 3000 amino acids, which can bind directly to DNA, by helix-loop-helix domain. This protein is important with *BRCA1* in homologous recombination repair (HRR) mechanism of double-strand DNA breaks. *BRCA2* interacts directly with RAD51 at the C-terminal region and seems to be also involved in its transport to the nucleus.

Hypoxia is a typical feature of microenvironment of several solid tumors and is associated with poor prognosis in several cancer types, including BC [37]. Under conditions of severe hypoxia, several cancer cell lines exhibit genetic instability showing downregulation of *MLH1* and *MSH2* expressions in a p53- and HIF-1*α*-dependent manner. Since tumor hypoxia is known to be an important factor for the expression of many genes involved in tumorigenesis, cell cycle regulation, and genetic instability [38], we performed a microarray-based gene expression analysis in order to determine different expression profiles in MCF-7, MDA-MB-231, and SKBr3 BC cells in response to hypoxia for 24 h and 48 h, respectively. In addition, cell proliferation assays by BrdU allowed us to hypothesize that hypoxia could negatively regulate the BC cell growth in short term in vitro studies. This microarray study allowed us to identify a set of 270 DEGs in hypoxia (81 downregulated and 189 upregulated) common to three BC cell lines (fold change > *|*0.5*|* and *P* < 0.05). Our attention was focused mainly on the genes showing a significant variation in expression levels involved in proliferation, cell cycle progression and regulation, mitosis, DDR mechanisms, and response to hypoxia. After hypoxia for 24 h and 48 h, the following main molecular pathways were found altered in BC cell lines: DNA damage repair, cell cycle regulation, HIF-1*α* network, and mitosis control. Among the DEGs involved in proliferation and cell cycle control, *CDKN1A* (p21^Cip1^), that can elicit the G1/S checkpoint, was upregulated, while genes that further determine entry from G1 to S-phase, including *CDC6*, *CCND1*, *CCNE2*, and *CDK2,* were decreased in expression. Concomitant with decreased G1 to S-phase progression, a reduced expression of genes that regulate the passage of cells through G2/M, including* CCNA2*, *CCNB1*, *CCNB2*, *CDC2 *(*CDK1*), and *CDC25C*, was also found in MCF-7 and SKBr3 cells exposed to hypoxia for 24 h and 48 h and in MDA-MB-231 cells under hypoxia for 24 h. In addition, among the DEGs involved in HIF-1a network, *VEGF-A*, *SLC2A1*, *JUN*, *FOS,* and other genes encoding for glycolytic enzymes (*PGK1*, *PFKFB3*, *HK2*, and *ALDOA*) were upregulated in all three analyzed BC cell lines. Finally, microarray analysis showed a significant downregulation of expression of the DEGs involved in DNA damage repair (DDR) pathways: mismatch repair (MMR), nucleotide excision repair (NER), nonhomologous end-joining (NHEJ), and homologous recombination repair (HRR). The most significant gene expression variations were observed in the HRR pathway. In particular, in HRR we found a significant decrease in expression levels of *BRCA1*, *BRCA2*, *RAD51* and *RAD50*. Also, other genes involved in MMR mechanism were found downregulated (*MLH1*, *MSH2,* and *MSH6*).

In a previous work, Meng et al. reported that hypoxia downregulates the expression of DNA double-strand break (DNA-dsb) repair genes involved in HHR mechanism, including *BRCA1* and *BRCA2*, in prostate cancer cell lines [39].

For the first time, our microarray study showed that hypoxia induces a noteworthy downregulation of *BRCA2 *expression that could involve an important pathophysiological role in BC. Quantitative real-time PCR and western blot analyses confirmed microarray results. *BRCA2* downregulation by hypoxia may represent an interesting mechanism of functional BRCA inactivation in the absence of genetic mutations. 

## 5. Conclusions

Since *BRCA2* is an important regulator of homologous recombination process in mammalian cells, its downregulation could play a critical role in DNA damage repair providing innovative approaches for the development of novel possible therapeutic strategies against BC. These findings may provide new insights into the mechanisms underlying genetic instability mediated by hypoxia and BRCA involvement in sporadic breast cancers. However, further studies will be needed to better understand the mechanisms underlying the hypoxia-induced BRCA downregulation.

## Supplementary Material

Table S1: Includes the differentially expressed genes involved in DNA damage repair (DDR) pathways in breast cancer cell lines under hypoxia. The Probe ID is the code provided by Affymetrix for each gene. All showed Signal values are in logarithm scale. The Signal value of each gene, for each experimental condition, is derived from the mean of two biological replicates. The Signal is the quantitative measure of the relative abundance of a transcript. N= Normoxia; H24= Hypoxia for 24 h; H48= Hypoxia for 48 h. Fold Change >|0.5| and P< 0.05.

## References

[1] Desantis C, Siegel R, Bandi P, Jemal A (2011). Breast cancer statistics, 2011. *CA Cancer Journal for Clinicians*.

[2] Apostolou P, Fostira F (2013). Hereditary breast cancer: the era of new susceptibility genes. *BioMed Research International*.

[3] Mavaddat N, Antoniou AC, Easton DF, Garcia-Closas M (2010). Genetic susceptibility to breast cancer. *Molecular Oncology*.

[4] Wilson CA, Ramos L, Villaseñor MR (1999). Localization of human BRCA1 and its loss in high-grade, non-inherited breast carcinomas. *Nature Genetics*.

[5] Birgisdottir V, Stefansson OA, Bodvarsdottir SK, Hilmarsdottir H, Jonasson JG, Eyfjord JE (2006). Epigenetic silencing and deletion of the BRCA1 gene in sporadic breast cancer. *Breast Cancer Research*.

[6] Murphy CG, Moynahan ME (2010). BRCA gene structure and function in tumor suppression: a repair-centric perspective. *Cancer Journal*.

[7] Jasin M (2002). Homologous repair of DNA damage and tumorigenesis: the BRCA connection. *Oncogene*.

[8] Bindra RS, Glazer PM (2005). Genetic instability and the tumor microenvironment: towards the concept of microenvironment-induced mutagenesis. *Mutation Research*.

[9] Fan R, Kumaravel TS, Jalali F, Marrano P, Squire JA, Bristow RG (2004). Defective DNA strand break repair after DNA damage in prostate cancer cells: implications for genetic instability and prostate cancer progression. *Cancer Research*.

[10] Koshiji M, To KK-W, Hammer S (2005). HIF-1*α* induces genetic instability by transcriptionally downregulating MutS*α* expression. *Molecular Cell*.

[11] Reynolds TY, Rockwell S, Glazer PM (1996). Genetic instability induced by the tumor microenvironment. *Cancer Research*.

[12] Hammond EM, Dorie MJ, Giaccia AJ (2003). ATR/ATM targets are phosphorylated by ATR in response to hypoxia and ATM in response to reoxygenation. *Journal of Biological Chemistry*.

[13] Coquelle A, Toledo F, Stern S, Bieth A, Debatisse M (1998). A new role for hypoxia in tumor progression: induction of fragile site triggering genomic rearrangements and formation of complex DMs and HSRs. *Molecular Cell*.

[14] Evans JW, Chernikova SB, Kachnic LA (2008). Homologous recombination is the principal pathway for the repair of DNA damage induced by tirapazamine in mammalian cells. *Cancer Research*.

[15] Semenza GL (2012). Cancer-stromal cell interactions mediated by hypoxia-inducible factors promote angiogenesis, lymphangiogenesis, and metastasis. *Oncogene*.

[16] Chen HHW, Su W-C, Lin P-W, Guo H-R, Lee W-Y (2007). Hypoxia-inducible factor-1*α* correlates with MET and metastasis in node-negative breast cancer. *Breast Cancer Research and Treatment*.

[17] Ben Lassoued A, Beaufils N, Dales JP, Gabert J (2013). Hypoxia-inducible factor-1alpha as prognostic marker. *Expert Opinion on Medical Diagnostics*.

[18] Nordsmark M, Bentzen SM, Rudat V (2005). Prognostic value of tumor oxygenation in 397 head and neck tumors after primary radiation therapy. An international multi-center study. *Radiotherapy and Oncology*.

[19] Dales J-P, Beaufils N, Silvy M (2010). Hypoxia inducible factor 1*α* gene (HIF-1*α*) splice variants: potential prognostic biomarkers in breast cancer. *BMC Medicine*.

[20] Chan N, Milosevic M, Bristow RG (2007). Tumor hypoxia, DNA repair and prostate cancer regression: new targets and new therapies. *Future Oncology*.

[21] Chaudary N, Hill RP (2007). Hypoxia and metastasis. *Clinical Cancer Research*.

[22] Yoshimura M, Itasaka S, Harada H, Hiraoka M (2013). Microenvironment and radiation therapy. *BioMed Research International*.

[23] Van Der Groep P, Bouter A, Menko FH, Van Der Wall E, Van Diest PJ (2008). High frequency of HIF-1*α* overexpression in BRCA1 related breast cancer. *Breast Cancer Research and Treatment*.

[24] Vaupel P (2004). The role of hypoxia-induced factors in tumor progression. *Oncologist*.

[25] Semenza GL (2000). Hypoxia, clonal selection, and the role of HIF-1 in tumor progression. *Critical Reviews in Biochemistry and Molecular Biology*.

[26] Mihaylova VT, Bindra RS, Yuan J (2003). Decreased expression of the DNA mismatch repair gene Mlh1 under hypoxic stress in mammalian cells. *Molecular and Cellular Biology*.

[27] Bindra RS, Gibson SL, Meng A (2005). Hypoxia-induced down-regulation of BRCA1 expression by E2Fs. *Cancer Research*.

[28] Federico M, Symonds CE, Bagella L (2010). R-Roscovitine (Seliciclib) prevents DNA damage-induced cyclin A1 upregulation and hinders non-homologous end-joining (NHEJ) DNA repair. *Molecular Cancer*.

[29] Gautier L, Cope L, Bolstad BM, Irizarry RA (2004). affy—analysis of Affymetrix GeneChip data at the probe level. *Bioinformatics*.

[30] Irizarry RA, Hobbs B, Collin F (2003). Exploration, normalization, and summaries of high density oligonucleotide array probe level data. *Biostatistics*.

[31] Smyth GK (2004). Linear models and empirical bayes methods for assessing differential expression in microarray experiments. *Statistical Applications in Genetics and Molecular Biology*.

[32] Benjamini Y, Drai D, Elmer G, Kafkafi N, Golani I (2001). Controlling the false discovery rate in behavior genetics research. *Behavioural Brain Research*.

[33] Wettenhall JM, Simpson KM, Satterley K, Smyth GK (2006). affylmGUI: a graphical user interface for linear modeling of single channel microarray data. *Bioinformatics*.

[34] Kanehisa M, Goto S (2000). KEGG: kyoto encyclopedia of genes and genomes. *Nucleic Acids Research*.

[35] Avni R, Cohen B, Neeman M (2011). Hypoxic stress and cancer: imaging the axis of evil in tumor metastasis. *NMR in Biomedicine*.

[36] van der Groep P, van Diest PJ, Smolders YH (2013). HIF-1alpha overexpression in ductal carcinoma in situ of the breast in BRCA1 and BRCA2 mutation carriers. *PLoS One*.

[37] Favaro E, Lord S, Harris AL, Buffa FM (2011). Gene expression and hypoxia in breast cancer. *Genome Medicine*.

[38] Kunz M, Ibrahim SM (2003). Molecular responses to hypoxia in tumor cells. *Molecular Cancer*.

[39] Meng AX, Jalali F, Cuddihy A (2005). Hypoxia down-regulates DNA double strand break repair gene expression in prostate cancer cells. *Radiotherapy and Oncology*.

